# Setting Research Priorities To Reduce Global Mortality from Childhood Diarrhoea by 2015

**DOI:** 10.1371/journal.pmed.1000041

**Published:** 2009-03-10

**Authors:** Olivier Fontaine, Margaret Kosek, Shinjini Bhatnagar, Cynthia Boschi-Pinto, Kit Yee Chan, Christopher Duggan, Homero Martinez, Hugo Ribeiro, Nigel C Rollins, Mohammed A Salam, Mathuram Santosham, John D Snyder, Alexander C Tsai, Beth Vargas, Igor Rudan

## Abstract

Olivier Fontaine and colleagues applied a priority-setting methodology to identify research priorities aimed at reducing global diarrhea mortality by 2015.

Childhood diarrhoea still claims nearly 2 million lives each year and remains responsible for 18% of all child deaths [1,2]. Regardless of this, research interest in this disease has been steadily decreasing after the development of cost-effective interventions in the 1980s [3]. In addition, the amount of available research funds per disability-adjusted life year (DALY) are several orders of magnitude lower for diarrhoea when compared to some other diseases, such as autism or diabetes type 2 [4]. The UN's Millennium Development Goal #4 (MDG4) states that childhood mortality should be reduced by two thirds between 1990 and 2015 [5], but recent estimates show that the progress in mortality reduction has not accelerated in comparison to 30 years ago [2]. Therefore this MDG target is likely to be missed. However, the reduction of child deaths by two thirds could be achieved by 2015 if presently available cost-effective interventions were delivered to those who need them most, and if there were sufficient financial resources to ensure their delivery [6,7].

Why is greater progress not being achieved? One of the key reasons is lack of knowledge on how to implement existing cost-effective interventions and on how to achieve greater coverage of these interventions in low-resource settings [8,9]. This gap in knowledge can only be filled by appropriately targeted research. To assist donors in understanding the potential of different research avenues to contribute to reducing the burden of disease and disability, the Child Health and Nutrition Research Initiative (CHNRI) of the Global Forum for Health Research recently developed a methodology that allows systematic listing and transparent scoring of many competing research options, thus exposing their strengths and weaknesses [10–12]. The Department of Child and Adolescent Health and Development (CAH) of the World Health Organization is currently using the CHNRI methodology to develop research priority issues on the major causes of child deaths. In this paper, we present the results of this research priority-setting process applied by CAH for childhood diarrhoea.

## The CHNRI Methodology for Priority Setting in Health Research Investments

The CHNRI methodology for setting priorities in health research investments was proposed as a tool that could be used by those who develop research policy and/or invest in health research [10–12]. It should assist them to understand (i) the full spectrum of research investment options, (ii) the potential risks and benefits that can result from investments in different research options, and (iii) the likelihood of achieving reductions of persisting burden of disease and disability through investments. The CHNRI methodology has three stages: input from investors and policy makers (defining the context and criteria for priority setting); input from technical experts (listing and scoring research investment options); and input from other stakeholders (weighing the criteria according to wider societal system of values) [10–12].

The aim of this particular implementation of the CHNRI method was to inform key global donors, investors in health research (especially of public funds), and international agencies on research investment policies that are expected to address MDG4 in the most effective way if the commitment to achieving this goal is genuine. In choosing this context, we did not mean to downplay the importance of any other issues, such as context-specific issues at local or regional levels, the large problem of diarrhoea morbidity, or any collateral beneficial effects of investments in diarrhoea research expected through improvement of malnutrition and other cross-cutting issues [13–15]. Several papers that will be informative on research priorities in those specific contexts have already been published [16] or are in preparation (I. Rudan, personal communication).

**Summary Points**

- This paper aims to define health research priority issues on childhood diarrhoea to improve the rate of progress in reducing global diarrhoea mortality by 2015, as set out in the UN's Millennium Development Goal #4.
- The authors applied the methodology for setting priorities in health research investments recently developed by CHNRI.
- The top 10% of research investment priorities were dominated by health systems and policy research questions and epidemiological questions, mainly targeted at better understanding the barriers towards implementation, effectiveness, and optimisation of use of available interventions.
- Improving the acceptability and effectiveness of oral rehydration solution and zinc for the treatment of diarrhoea were ranked first.
- The implementation of the CHNRI methodology showed that, within the context of MDG4, a better balance should be achieved between investments in specific domains of health research at the global level.

**Figure 1:**
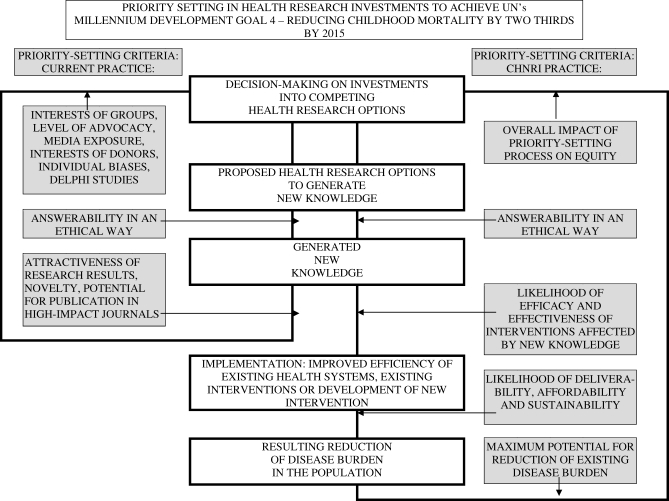
CHNRI's Conceptual Framework This framework shows key steps required to get from investments in health research options to decrease in burden of death, disease, or disability. The framework identifies criteria that are important for discriminating among competing research options and judging the likelihood that they will achieve their goals: (i) answerability; (ii) effectiveness; (iii) deliverability; (iv) maximum potential for disease burden reduction; and (v) predicted impact on equity in the population (right side). These criteria are not necessarily what drive investment decisions in health research today (left side).

Based on CHNRI's simple conceptual framework (Figure 1), five criteria were agreed upon: (i) answerability (in an ethical way); (ii) likelihood of effectiveness; (iii) likelihood of deliverability, affordability, and sustainability; (iv) maximum potential impact on burden reduction; and (v) predicted impact on equity. The detailed CHNRI methodology process is shown in Text S1. The process yielded an initial list of 154 research questions. The exact scores given to all 154 research questions from individual experts are presented in Table S1. The final list of priorities with intermediate and final priority scores for all 154 research questions is presented in Table S2. The full list of technical experts who were invited to participate, their expertise, and reasons for non-participation from those who declined are presented in Table S3.

## Results

Table 1 and Table 2 show the top and bottom 10% of the 154 research questions. Both tables clearly present the likelihood for each research question to comply with each of the five chosen priority-setting criteria. Research questions from all four broad research domains (epidemiological research; health systems and policy research; research to improve the existing interventions; and research to develop new interventions) feature in both the top 10% and the bottom 10% of research questions. In Table 1, research questions with ranks 1, 2, 8, 9, 11, and 12 represent the domain of epidemiological research; 3, 4, 5, 6, and 10 represent health systems and policy research; 13 and 14 represent research to improve the existing interventions; and 7 and 15 represent research to develop new interventions. In Table 2, the same is true for questions with ranks 141 and 143 (epidemiological research); 147 and 148 (health systems and policy research); and 146 (research to improve the existing interventions); while the remaining questions represent the “research to develop new interventions” domain. This suggests that the CHNRI method managed to compare and discriminate among questions addressing very different domains of health research using the same framework, and that there was no systematic bias against research questions from any of the four domains.

**Table 1:**
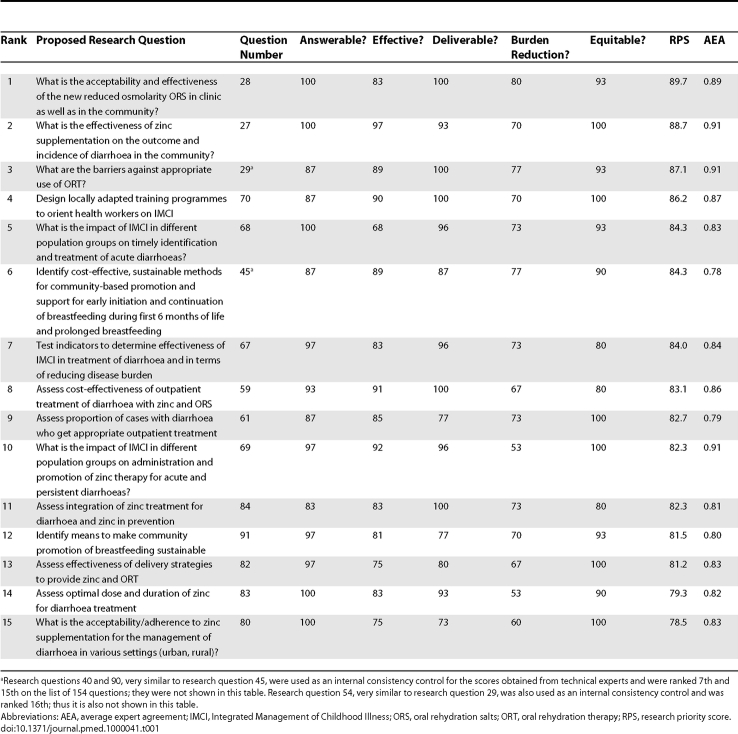
The 15 Research Questions That Achieved the Highest Overall Research Priority Score with Average Expert Agreement Related To Each Question

**Table 2:**
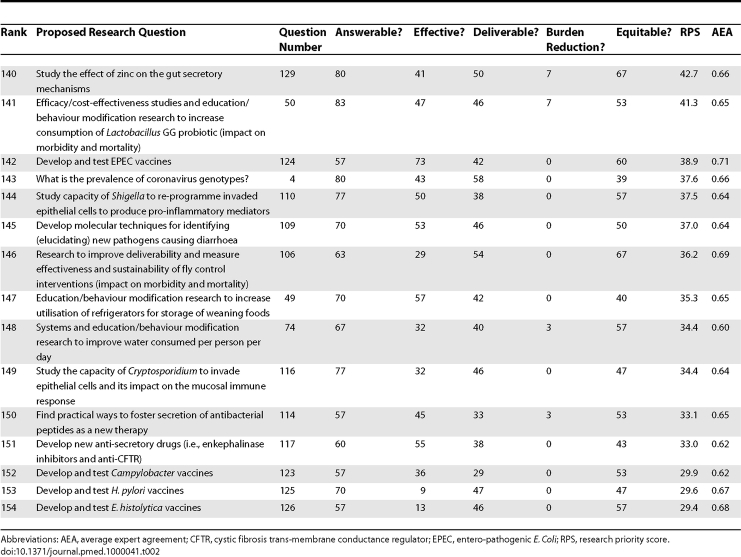
The 15 Research Questions That Achieved the Lowest Overall Research Priority Score with Average Expert Agreement Related To Each Question

A look at the top 20% research questions reveals a predominance of research questions from the domains of “health systems and policy research” (13/31) and “epidemiological research” (11/31), while a smaller number came from the domains of “research to develop new interventions” (4/31) or “research to improve the existing interventions” (3/31). This is not surprising because technical experts were asked to define research priorities that could lead to notable improvements in reduction of diarrhoea mortality by the year 2015. This short time frame benefited epidemiological questions that proposed to assess and confirm the value of existing and available cost-effective interventions in different contexts (such as oral rehydration solutions, zinc supplementation, exclusive breastfeeding, and integrated management of childhood illness). It also highlighted the value of investments in health systems and policy research that proposed to identify key obstacles to delivery, affordability, and sustainability of implementation of those interventions on a larger scale. The scores also recognised the value of research that aimed to improve and optimise the use of those interventions (alone or in combination) in different contexts, and to develop entirely new interventions and approaches that could assist delivery or acceptance of the existing cost-effective interventions.

Among the bottom 20% of research options, the majority (18/31) proposed development of entirely new interventions. Again, this is not surprising given the specified time frame (the year 2015). In addition, eight issues from the domain of “epidemiological research”, three from the domain of “health systems and policy research”, and two from the domain of “improvement of existing interventions” were not seen by the scorers as priority. In the large majority of cases, the main reason for this was minimal, or entirely non-existent, optimism towards their possible impact on reduction of diarrhoea mortality within the context defined above (i.e., by 2015). This was coupled with concerns over effectiveness of many of the proposed new interventions, such as developing and testing vaccines for Helicobacter pylori and Entamoeba histolytica, heating weaning foods using solar-powered ovens, or investing in improvement of fly control interventions. Another prevalent concern common among many low-scoring options was that they would be more likely to increase inequity rather than decrease it, at least by 2015. For example, new interventions are very likely to be initially available only to those who can afford them.

Good discrimination between the levels of agreement among the scorers on the priority of the 154 questions was achieved by calculating “average expert agreement” (see Text S1). The scores ranged from 0.55 to 0.91, indicating the proportion of scorers that gave the same most frequent answer to an average question they were asked in relation to a specific research investment option. Average expert agreement values are also presented for the top and bottom 10% of research questions in Tables 1 and 2. Generally, the questions over which the greatest level of overall agreement was observed among the experts were those that also achieved very high overall research priority scores. The greatest points of controversy were the research questions related to development of non-existing vaccines, entirely new interventions, and education/behaviour modification research.

## Discussion

The amount of funding available today for health research globally is unprecedented—the research investment market has been growing steadily over the past decade to more than US$126 billion in 2003 [4]. However, large inequities exist between amounts invested in different conditions that contribute to the global burden of disease. For example, while research on diabetes type 2 receives approximately US$102 per DALY, research on diarrhoea receives less than US$10 per DALY [4].

Perhaps a more pressing issue is the way in which investors manage their risk of investing in different health research domains. The risk is highly dependent on the context and degree of urgency to identify interventions for particular diseases. While high-risk high-profit investment strategies (e.g., long-term strategic investments in basic research) may be justified in cases of chronic diseases, which can already be controlled by changes in diet and lifestyle and do not cause imminent threat to life, the situation with childhood diseases such as diarrhoea and pneumonia is quite different. Those two diseases combined cause more child deaths each year worldwide than annual deaths attributable to smoking in all ages, or twice as many annual deaths as HIV/AIDS globally [4]. The persisting high mortality from diarrhoea in the presence of existing cost-effective interventions and available resources to implement them represents a continuing scandal. Given the consequences of the disease in terms of persisting child mortality, the level of urgency in dealing with this problem is very different than for other chronic diseases that contribute heavily to DALYs. We believe that this should be reflected in health research policies and investment strategies of the major donors.

Investment in global health research today would benefit from consensus regarding the context, appropriate investment strategies, and co-ordination to achieve significant reduction of the disease burden in the foreseeable future. The present exercise was designed to assist investors and policy makers in making more informed choices on their investments in health research on diarrhoea by making apparent the risks and potential benefits associated with investments in a broad spectrum of health research options. The expected “profit” from investments is associated with generating new knowledge that can be translated into development of new (or improvement of existing) interventions, which are effective, deliverable, affordable, and can reduce the existing burden of disease and disability in an equitable way. The risk is associated with research that is not likely to be answerable, or that develops products unlikely to be effective, deliverable, affordable, or sustainable by those who need them most. Investors' preference for high-risk investment in health research is particularly questionable when it is occurring in a context that requires urgent progress, such as childhood diarrhoea. The focus on complex challenges of implementation (i.e., improving health systems, training health workers including poorly educated village health workers, improving drug supply and delivery at community level, etc.), which the exercise highlighted, was reflected in many research questions being ranked near the top of the list of overall priorities.

The implementation of the CHNRI methodology showed that, within the context of MDG4, a better balance should be achieved between specific domains of health research. Along with continuing strategic long-term investments in vaccines and other new interventions, which represent high-risk high-profit strategies, the CHNRI process suggested that more attention should be given to health policy research, health systems research, operations research, and research that addresses political, economic, social, cultural, behavioural, and infrastructure issues surrounding the problem of child mortality. These domains of health research are rarely recognised as attractive by investors in health research because their results are unlikely to grab the headlines or be published in journals with high impact factors or lead to patents and commercial products. Yet, they can generate new knowledge that can be very helpful in achieving real progress in disease burden reduction. The identified priorities are also in good agreement with the research supported by CAH at present. They emphasise the evaluation of existing interventions and the development and testing of new delivery approaches of existing interventions. They also highlight the value of research on preventive measures (breastfeeding, rotavirus vaccination, measles vaccination, etc.), with research on new interventions being downplayed within the real context.

## Advantages and Limitations of the CHNRI Methodology

Although the advantages of the CHNRI methodology represent a serious attempt to deal with many issues inherent to a highly complex process of research investment priority setting, there are still concerns over the validity of the CHNRI approach and related biases. One of them is related to the fact that many possible good ideas (“research investment options”) may not have been included in the initial list of research options that was scored by the experts, and to the potential bias towards items that get the greatest press. Another concern over the CHNRI process is that its end product represents a possibly biased opinion of a very limited group of involved people. In theory, a chosen group of experts can have biased views in comparison to any other potential groups of experts. Those limitations are described and discussed in greater detail in Text S1.

## Conclusions

The main message of the process is that the research priorities to reduce global mortality from childhood diarrhoea within the present context are dominated by health systems, policy research, and epidemiological questions. These questions are mainly targeted at better understanding the barriers towards implementation, effectiveness, and optimisation of use of available interventions and programmes such as oral rehydration solution, zinc supplementation, exclusive breastfeeding, and integrated management of childhood illness. If progress towards reduction of global diarrhoea mortality is to be improved by 2015, these are the research questions that are most likely to be of greatest importance. However, very few donors agencies recognise the importance of these domains of health research and are willing to readily invest in those options [4,17]. The core group of CHNRI experts made several serious attempts to influence the key donors and point to this gap and serious imbalance in health research investing between “upstream” and “downstream” health research. This exercise is the best example to date conducted at the global level.

## Supporting Information

Text S1 CHNRI Methodology (69 KB DOC).

Table S1 Scores Assigned to Each of the 154 Research Questions by Technical Experts (183 KB XLS).

Table S2 Final List of Research Questions Ranked According To Weighted Research Priority Scores (72 KB XLS).

Table S3 An Overview of Expert Selection and Their Choice To Participate (or Decline Participation) All participation in this particular CHNRI exercise was voluntary and carried out without specific funding support.
(32 KB DOC).

## Abbreviations

CAH: Department of Child and Adolescent Health and Development of the World Health Organization
CHNRI: Child Health and Nutrition Research Initiative
DALY: disability-adjusted life year
MDG: Millennium Development Goal
