# Supplementary material for: Setting Research Priorities To Reduce Global Mortality from Childhood Diarrhoea by 2015
Source: PLoS Med. 2009 Mar 10;6(3):e1000041. doi: 10.1371/journal.pmed.1000041 (PMC2653551; doi:10.1371/journal.pmed.1000041)
Supplement: Supplementary file 1 [file pmed.1000041.sd001.doc]

### **Supplementary File Text S1.**

### **CHNRI Methodology**

### **Defining the context and criteria for priority setting**

### Specifying the context a priori is a critical part of the CHNRI process, because priority scores for many research investment options may change substantially according to different contexts. The context for this exercise was defined to address research investment priorities that could assist in achieving UN's MDG4. This is a goal on which global consensus has been reached and wide political commitment has been made.

The context was specified by the WHO/CAH as follows:

- **Burden of disease of interest:** deaths from diarrhoea;
- **Population of interest:** children under 5 years of age in all developing countries, where nearly all cases of diarrhoea deaths occur;
- **Existing policy/target:** reduction of diarrhoea mortality by two thirds by 2015 (in order to contribute to the achievement of the UN’s MDG4)
- **Level of urgency:** high (because the goal is not being achieved)
- **Time frame:** to achieve detectable improvement in the rate of diarrhoea mortality reduction by 2015;

**Choice of technical experts, systematic listing and scoring of research investment options**

The co-ordinator of the project for WHO/CAH (OF) invited a group of 24 international technical experts with interest in diarrhoeal diseases to participate in the CHNRI process. The selection of experts was based primarily on their track record of conducting research of high quality for many years on the topic of childhood diarrhoea in developing countries. Every effort was made to invite a mix of people with different backgrounds (clinicians, epidemiologists, public health experts, programme leaders and basic scientists) and from different countries (both developed and developing ones), so that the mix contains a diversity of views from the wider research community. In addition, every expert was only allowed to score 2 criteria of their choice, on which they felt more knowledgeable, thus limiting each individual expert's potential impact on overall scores.

The first task of the technical experts was to propose a large spectrum of research questions in a systematic way, according to the framework developed by CHNRI (**Table T1**). The conceptual framework for this process was described in detail elsewhere [1]. The co-ordinator from WHO/CAH collected all the proposed ideas from each of the experts independently by e-mail. The process was open-ended and it initially yielded several hundreds research questions. Then the list of research questions was consolidated and narrowed down to a manageable size for the scorers. In producing this list, the co-ordinator limited the overlap between proposed ideas and ensured that the research questions were phrased in a way that would make the expected new knowledge apparent. We feel that the final list of 154 questions covers the wide spectrum of all possible questions

The second task of the experts was to score all research questions independently, according to the five agreed criteria. For each of the 154 research questions and each criterion, each expert answered three questions targeted to assess the likelihood of the proposed research to comply with the priority-setting criterion (see attached Box 1). This task was completed by thirteen experts, and the process was considered complete when each of the 5 criteria was scored by at least five technical experts. The entire process was conducted and completed via e-mail between October 2006 and October 2007. Further information on methods related to this part of the priority-setting process were presented elsewhere in greater details [1-4].

**Box 1**

Questions answered by technical experts to assign intermediate scores to competing research options. (possible answers: Yes=1; No=0; Informed but undecided answer: 0.5; Not sufficiently informed: blank)

CRITERION 1: Likelihood that research would lead to new knowledge (enabling a development / planning of an intervention) in ethical way.

1. Would you say the research question is well framed and endpoints are well defined?

2. Based on: (i) the level of existing research capacity in proposed research; and (ii) the size of the gap from current level of knowledge to the proposed endpoints; would you say that a study can be designed to answer the research question and to reach the proposed endpoints of the research?

3. Do you think that a study needed to answer the proposed research question would obtain ethical approval without major concerns?

CRITERION 2: Assessment of likelihood that the intervention resulting from proposed research would be effective.

1. Based on the best existing evidence and knowledge, would the intervention which would be developed / improved through proposed research be efficacious?

2. Based on the best existing evidence and knowledge, would the intervention which would be developed / improved through proposed research be effective?

3. If the answers to either of the previous two questions is positive, would you say that the evidence upon which these opinions are based is of high quality?

CRITERION 3: Assessment of deliverability, affordability and sustainability of the intervention resulting from proposed research.

1. Taking into account the level of difficulty with intervention delivery from the perspective of the intervention itself (e.g. design, standardization, safety), the infrastructure required (e.g. human resources, health facilities, communication and transport infrastructure) and users of the intervention (e.g. need for change of attitudes or beliefs, supervision, existing demand), would you say that the endpoints of the research would be deliverable within the context of interest?

2. Taking into account the resources available to implement the intervention, would you say that the endpoints of the research would be affordable within the context of interest?

3. Taking into account government capacity and partnership requirements (e.g. adequacy of government regulation, monitoring and enforcement; governmental intersectoral coordination, partnership with civil society and external donor agencies; favourable political climate to achieve high coverage), would you say that the endpoints of the research would be sustainable within the context of interest?

CRITERION 4: Assessment of maximum potential of disease burden reduction.

*As this dimension is considered "independent" of the others, in order to score competing options fairly, their maximum potential to reduce disease burden should be assessed as potential impact fraction under an ideal scenario, i.e., when the exposure to targeted disease risk is decreased to 0% or coverage of proposed intervention is increased to 100% (regardless of how realistic that scenario is at the moment - that aspect will be captured by other dimensions of priority setting process, such as deliverability, affordability and sustainability)*

*Non-existing interventions**

*Maximum potential to reduce disease burden should be computed as "potential impact fraction” for each proposed research avenue, using the equation:*

*PIF = [S_(i=1 to n)_ P_i_ (RR_i_-1)] / [S_(i=1 to n)_ P_i_ (RR_i_-1) + 1];*

*where PIF is “potential impact fraction” to reduce disease burden through reducing risk exposure in the population from the present level to 0% or increasing coverage by an existing or new intervention from the present level to 100%; RR is the relative risk given exposure level (less than 1.0 for interventions, greater than 1.0 for risks), P is the population level of distribution of exposure, and n is the maximum exposure level.*

*Existing interventions***

*Maximum potential to reduce disease burden should be assessed from the results of conducted intervention trials; if no such trials were undertaken, then it should be assessed as for non-existing interventions.*

*Then, the following questions should be answered:*

1. Taking into account the results of conducted intervention trials**, or for the new interventions the proportion of avertable burden under an ideal scenario*, would you say that the successful reaching of research endpoints would have a capacity to remove 5% of disease burden or more?

2. To remove 10% of disease burden or more?

3. To remove 15% of disease burden or more?

CRITERION 5: Assessment of the impact of proposed health research on equity.

1. Would you say that the present distribution of the disease burden affects mainly the underprivileged in the population?

2. Would you say that either (i) mainly the underprivileged, or (ii) all segments of the society equally, would be the most likely to benefit from the results of the proposed research after its implementation?

3. Would you say that the proposed research has the overall potential to improve equity in disease burden distribution in the long term (e.g. 10 years)?

**Community involvement - input from larger group of stakeholders**

CHNRI methodology ensures community involvement through incorporating the opinions and values from a broader group of stakeholders (e.g. expected recipients of the research, taxpayers who fund health research, health workers, journalists and media, experts in ethics, law, political science, etc.) [5]. Stakeholders lack expertise to directly decide research priorities, but their opinions and values can still be incorporated by weighing the chosen priority-setting criteria according to their perceived importance. In three separate exercises that took place between March and June 2006, CHNRI consultants interviewed three different groups of stakeholders [5]. We decided to use weights provided by the group of stakeholders most appropriate to this exercise (members of an international priority setting network co-ordinated from the University of Toronto) to compute the overall priority score for each of the 154 research options. More detailed explanations on the rationale and methods for including stakeholders' opinions in the process are presented elsewhere [5].

**Computations of "research priority scores"**

All the experts answered the questions listed in **Box 1** by ‘Yes’ (1 point) or ‘No’ (0 points). They were also allowed to declare an informed but undecided answer (0.5 points) or declare themselves insufficiently informed to answer the question (missing input). Thus, the proposed research questions got a score for each of the five criteria as "the proportion of maximum possible points scored when an answer was given" (i.e., excluding the missing input). They represent a direct measure of **collective optimism** of the scorers. Each of the 154 listed research questions received five intermediate scores (each ranging between 0%-100 %), which were then weighted according to the input from the stakeholders. The weights were applied as follows: a weight of 1.75 was given to the criterion "maximum potential for disease burden reduction"; 0.96 to "answerability in an ethical way"; 0.91 to "predicted effect on equity in the population"; 0.89 to "deliverability, affordability and sustainability"; and 0.86 to the criterion "potential contribution to effectiveness" [5]. The overall research priority score (RPS) was then computed as the weighted mean of all five intermediate priority scores. The exact scores given to all 154 research questions from individual experts are presented in **supplementary file Table S1**. The final list of priorities with intermediate and final priority scores for all 154 proposed research questions is presented in supplementary file **Table S2.** The full list of technical experts who were invited to participate, their expertise and reasons for non-participation for those who declined are presented in **s**upplementary file **Table S3.**

**Assessment of agreement between scorers**

CHNRI methodology has the ability to expose the issues of greatest agreement and controversy. This allows more focused discussion among experts following this exercise, and informs the investors and policy makers about the amount of controversy that surrounds each research question. The datasets that CHNRI methodology produces are not appropriate for application of the usual Kappa agreement statistics, which has been discussed in detail elsewhere [6,7]. For each research investment option, we reported the average proportion of scorers that agreed on the 15 questions asked. This is computed for each scored research investment option as:

1	_15_ N (scorers who provided most frequent response)

AEA (average expert agreement) = ---- x Σ -------------------------------------------------

15	^q=1^	N (scorers who provided any response)

(where q is a question that experts are being asked to evaluate competing research investment options, ranging from 1 to 15).

For each evaluated research investment option, AEA is informing us, for an average question, what proportion of scorers gave the same most frequent answer. This parameter satisfactorily accounts for missing answers, is unaffected by responses of ‘undecided’, and is also unaffected by the varying number of scorers per criterion and differences in scorer composition for the different criteria.

**Advantages and limitations of the CHNRI methodology**

The applied CHNRI methodology proved to be helpful to systematically list and score a very large number of specific research questions, as shown recently in exercises conducted at national level in South Africa [4], and at global level for mental health research issues [8] and zinc deficiency [9]. Other advantages of the CHNRI process include its systematic nature, transparency, well defined (*a priori*) context and criteria chosen for discriminating between research investment options, a highly structured way in which relevant information is obtained from the scorers, independent scoring that limits influence of strong-minded individuals on the rest of the scorers, its informative and intuitive quantitative outputs and ability to expose points of greatest agreement and controversy.

Still, the methodology is not free of several possible biases. Although the advantages mentioned above represent a serious attempt to deal with many issues inherent to a highly complex process of research investment priority setting, there are still concerns over the validity of the CHNRI approach and related biases. One of them is related to the fact many possible good ideas (“research investment options”) may not have been included in the initial list of research options that was scored by the experts, and to the potential bias towards items that get the greatest press. The spectrum of research investment options listed initially in this exercise was derived through a systematic process, but it is not endless and it cannot ever cover every single research idea. Specific research methodologies (i.e. randomized clinical trials, etc.) are not mentioned because the research questions listed in that exercise are unlikely to be answered by a single well-defined study. Therefore, the CHNRI process aims to achieve reasonable coverage of the spectrum of possible ideas. After the completion of the exercise, approximate scores and ranks for some specific research questions that are missing in the initial systematic list could still be estimated – either by relating them to the most similar questions on the list or by having those missed questions scored by a single expert (or by a group), using the CHNRI framework and then comparing the computed score to all other scores received for the originally listed research options.

Another concern over the CHNRI process is that its end product represents a possibly biased opinion of a very limited group of involved people. In theory, a chosen group of experts can have biased views in comparison to any other potential groups of experts. However, the number of people globally who possess enough experience, expertise and knowledge on this issue (childhood diarrhoea) to be able to judge a very diverse spectrum of research questions is rather limited (although certainly much larger than the group that we eventually selected). If one thinks of this “global pool of technical experts” as the whole population that could theoretically be used to solicit expert opinion on the questions that need to be asked, we then selected a “sample” from that population, based on their track record in research on childhood diarrhoea. Given that the “sample” of the experts chosen for this exercise was one of the largest and the most diverse to ever conduct a CHNRI exercise to date, while the number of experts in this neglected health problem globally is not large, we doubt that there would be considerable differences in the composition of the initial list of questions (or results of the scoring process) if some other group of experts had been selected.

Obviously, CHNRI methodology is not free of bias that results from the choice of the experts, and different groups of experts may indeed have quite different opinions. However, the larger and more diverse the group of chosen experts (as in this case), the less likely is that the results of their scoring would significantly deviate from the output of any other large and diverse expert group, chosen from a limited “pool of global technical experts on childhood diarrhoea”.

# References

1. Rudan I, Chopra M, Kapiriri L, Gibson J, Lansang MA, et al. (2008) Setting priorities in global child health research investments: Universal challenges and conceptual framework. Croat Med J 49: 398-408.

2. Rudan I, El Arifeen S, Black RE. (2006) A systematic methodology for setting priorities in child health research investments. In: A new approach for systematic priority setting. Dhaka: Child Health and Nutrition Research Initiative. Available at: <http://www.chnri.org/section/publications>. Accessed 31 July 2008.

3. Rudan I, El Arifeen S, Black RE, Campbell H. (2007) Childhood pneumonia and diarrhoea: setting our priorities right. Lancet Inf Dis 7: 56-61.

4. Tomlinson M, Chopra M, Sanders D, Bradshaw D, Hendricks M, et al. (2007) Setting Priorities in Child Health Research Investments for South Africa. PLoS Med 4(8): E259.

5. Kapiriri L, Tomlinson M, Gibson J, Chopra M, El Arifeen S, et al. (2007) Child Health and Nutrition Research Initiative (CHNRI): Setting priorities in global child health research investments: Addressing the values of the stakeholders. Croat Med J 48: 618-627.

6. Cicchetti DV, Feinstein AR. (1990) High agreement but low Kappa: II. Resolving the paradoxes. J Clin Epidemiol 43: 551-8.

7. Tomlinson M, Rudan I, Saxena S, Swartz L, Tsai AC, et al. (2009) Setting investment priorities for research in global mental health. Bull World Health Organ (in press).

8. Chisholm D, Flisher AJ, Lund C, Patel V, Saxena S, et al. (2007) Scale up services for mental disorders: a call for action. Lancet 370: 1241-52.

9. Brown KH, Hess SY, Boy E, Gibson RS, Horton S, et al. (2008) Setting priorities for zinc-related health research to reduce children’s disease burden worldwide: an application of the CHNRI research priority-setting method. Public Health Nutr Apr 22: 1-8. [Epub ahead of print]

**Table T1:** CHNRI’s starting framework from which listing of many research options (level of 3-5-year research programmes) and research questions (level of individual research papers) were being proposed by technical experts to develop a consolidated list of 154 research questions.

| RESEARCH INSTRUMENT | RESEARCH AVENUE | RESEARCH OPTION | RESEARCH QUESTION |
| --- | --- | --- | --- |
| Epidemiological research | Measuring the burden | (Technical experts were invited to use categorization of research avenues and instruments to systematically propose a number of ‘research options’ within each of the avenues; ‘research options’ correspond to the level of 3-to-5-year research programmes) | (Technical experts were invited to propose a number of very specific ‘research questions’, corresponding to the title of individual research papers, within each of the ‘research avenues; eventually, after consolidation and removing of duplicate ideas, 154 such questions were retained for scoring) |
|  | Understanding risk factors |  |  |
|  | Evaluating the existing interventions |  |  |
| Health policy and systems research | Studying capacity to reduce exposure to proven health risks |  |  |
|  | Studying capacity to deliver efficacious interventions |  |  |
| Research to improve existing interventions | Research to improve deliverability |  |  |
|  | Research to improve affordability |  |  |
|  | Research to improve sustainability |  |  |
| Research for development of new interventions | Basic research |  |  |
|  | Clinical research |  |  |
|  | Public health research |  |  |
