# Supplementary material for: Setting Research Priorities To Reduce Global Mortality from Childhood Diarrhoea by 2015
Source: PLoS Med. 2009 Mar 10;6(3):e1000041. doi: 10.1371/journal.pmed.1000041 (PMC2653551; doi:10.1371/journal.pmed.1000041)
Supplement: Supplementary file 2 [file pmed.1000041.st003.doc]

**Supplementary File Table S3**

An overview of expert selection and their choice to participate (or decline participation). All participation in this particular CHNRI exercise was voluntary and carried out without specific funding support.

All the experts who were invited to participate in that exercise had a well known track record on research on diarrhoeal diseases, either as clinicians, epidemiologists, social scientists or public health specialists. However, we missed the group representing the rotavirus research (D.Steele and R. Glass).

A. "Experts that have participated in the exercise"

1. C. Duggan (clinician)

2. H. Ribeiro (clinician)

3. J. Snyder (epidemiologist, clinician and public health expert)

4. B. Vargas (social scientist and public health expert)

5. M. Kosek (epidemiologist)

6. MA Salam (clinician)

7. S. Bhatnagar (clinician)

8. N. Rollins (clinician and epidemiologist)

9. H. Martines (epidemiologist and public health expert)

10. M. Santosham (clinician, vaccine expert and public health expert)

11. C. Boschi-Pinto (epidemiologist and public health expert)

12. I. Rudan (epidemiologist, public health expert and basic scientist)

13. O. Fontaine (clinician, public health expert and basic scientist)

B. "Experts who did not participate after having been contacted"

1. C. Victora, epidemiologist and public health expert (said he had already participated in a similar pilot exercise on diarrhea in early phase of CHNRI methodology development, when he provided the scores for a limited set of questions)

2. C. Lanata (said he had led a similar pilot exercise on diarrhea in early phase of CHNRI methodology development, when he provided the scores for a limited set of questions)

3. Z. Bhutta, clinician and epidemiologist (said he had already participated in a similar pilot exercise on diarrhea in early phase of CHNRI methodology development, when he provided the scores for a limited set of questions)

4. D. Steele, vaccine specialist (accepted to participate but got too busy to go through the scoring; he provided comments on the identified research issues; comments that were incorporated in the final list of research issues that were scored)

5. R. Glass, vaccine specialist (moved from Center for Disease Control in Atlanta to become the head of the Fogarty Center at NIH- Washington)

6. Y. Gamatie, public health expert (apologized and said he could not contribute due to large commitments to some other projects)

7. M. Nichter, social scientist (responded that the process was too time consuming for him to contribute)

8. D. Mahalanabis, public health expert (never responded to repeated invitations)

9. Mk Bhan, clinician, epidemiologist and public health expert (never responded to repeated invitations)

10. D. Sack, public health expert (never responded to repeated invitations)

11. E. Salazar-Lindo, clinician (never responded to repeated invitations)

12. J Keusch, scientist (never responded to repeated invitations)
